# Benzoylaconine: Potential Therapeutic Agent for Cardiovascular Diseases From Fuzi

**DOI:** 10.1155/2024/4878103

**Published:** 2024-07-03

**Authors:** Chenggang Xu, Le Tang, Lixin Hu, Yunzhe Huang, Jin Tang, Xiaohu Wang, Feng Wang

**Affiliations:** ^1^ Department of Cardiology XuanCheng City Central Hospital, Xuancheng, China; ^2^ Pharmacology College Anhui Xinhua University, Hefei, China; ^3^ Department of Pharmaceutics China Pharmaceutical University, Nanjing, China; ^4^ Graduate School Wannan Medical College, Wuhu, China

**Keywords:** anti-inflammation, benzoylaconine, cardiac diseases, pharmacokinetics, pharmacological effects

## Abstract

Modern pharmacological studies have elucidated the presence of aconitine (AC) alkaloids, polysaccharides, and saponins as the primary bioactive constituents of Fuzi. Among these, benzoylaconine, a pivotal active compound, demonstrates notable pharmacological properties including antitumor, anti-inflammatory, and cardiovascular protective effects. In recent years, benzoylaconine has garnered significant attention in basic research on heart diseases, emerging as a focal point of investigation. This paper presents a comprehensive review of the pharmacological effects of benzoylaconine, alongside an overview of advancements in metabolic characterization. The objective is to furnish valuable insights that can serve as a cornerstone for further exploration, utilization, and advancement of benzoylaconine in pharmacological research.

## 1. Introduction

*Radix Aconiti lateralis praeparata*, known as “Fuzi” in Chinese, is the processed cotyledon root of *Aconitum carmichaeli*. It is traditionally valued and used for dispelling cold, relieving pain effects, and treating shock [1, 2]. It has been documented in the ancient Chinese pharmacopeia, the *Shennong Ben Cao Jing*, and is predominantly cultivated in several Chinese provinces, including Sichuan, Shanxi, Hubei, Hunan, and Yunnan. In traditional Chinese herbal medicine, Fuzi occupies an important position and is highly regarded for its significant pharmacological effects on the cardiovascular system. Specifically, it is frequently used in the treatment of hypotension, coronary artery disease, and shock resulting from acute myocardial infarction, particularly in cases of heart failure [3–5].

This medicinal plant contains a plethora of structurally distinct active ingredients, including aconitine (AC) alkaloids, polysaccharides, saponins, flavonoids, fatty acids, and sterols, which contribute to its pharmacological properties. Research has demonstrated that Fuzi exhibits a range of beneficial effects, including cardiomyocyte protection, antiarrhythmic, anti-inflammatory, and analgesic properties [6]. Additionally, it has shown promising antitumor properties [7, 8], leading to its widespread utilization in clinical practice [9, 10].

However, it is crucial to acknowledge that the use of Fuzi comes with inherent risks of toxic side effects. While Fuzi holds therapeutic potential in treating cardiac diseases, rheumatic diseases, tumors, and more at low doses, high doses may lead to adverse effects such as ventricular tachyarrhythmias, cardiac arrest, and even lethality [11]. Therefore, there is a pressing need for the development of safe, effective, and low-toxicity compounds derived from Fuzi to maximize its therapeutic benefits while minimizing potential risks.

**Figure 1:**
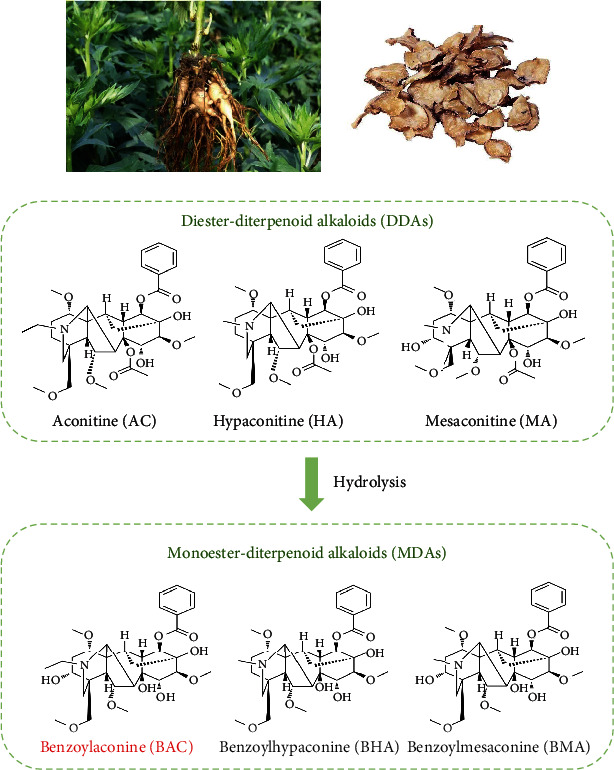
Relationships between the studied MDAs and DDAs of Fuzi.

The alkaloids present in Fuzi predominantly consist of monoesteric diterpene alkaloids (MDAs) and diester-type diterpenoid alkaloids (DDAs) [12], as depicted in Figure 1. These diterpenoid alkaloids not only serve as the essential active compounds responsible for Fuzi's medicinal effects but also constitute its toxic components [13].

Benzoylaconitine (BAC) is a monoester alkaloid and a characteristic constituent of Fuzi, but it is present in low levels. It is mainly produced by hydrolyzing the diester alkaloids during the decoction of Fuzi [14, 15]. This hydrolysis process effectively diminishes the toxicity of Fuzi, suggesting that BAC holds promise as a cardioprotective agent.

Based on an extensive review of existing literature, this paper focuses on consolidating, structuring, and presenting the most recent advancements regarding BAC in the realms of cardiovascular diseases, anti-inflammatory effects, and analgesic properties. The objective of this endeavor is to aid scientists and clinicians in comprehending the significance of BAC, thereby providing valuable insights to guide future research, development, and clinical utilization of this compound.

## 2. Literature Search Strategies on BAC

The EMBASE, China National Knowledge Infrastructure (CNKI), and PubMed databases were searched (up to Jan 2024) using the following terms: “Benzoylaconine” AND (“Isaconitine” OR “Pikraconitin” OR “pharmacokinetic” OR “Toxicity” OR “Pharmacological” OR “Cardiovascular”). The reference lists from the relevant studies were analyzed for additional literature.

## 3. Basic Properties and Information

**Figure 2:**
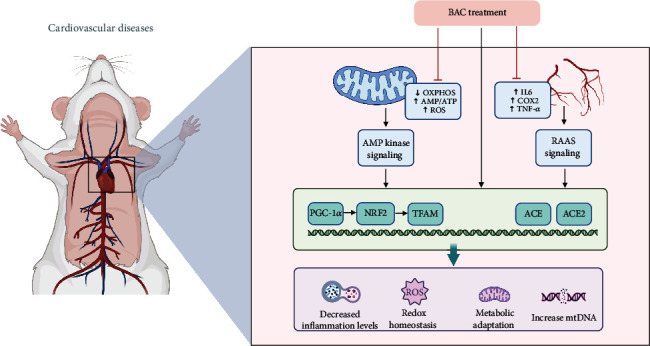
Mechanism of action of BAC on cardiovascular diseases.

BAC, a principal monoester alkaloid present in Fuzi, holds significance in traditional Chinese medicine. The 2020 edition of the *Chinese Pharmacopoeia* stipulates that the combined content of BAC, benzoylhypaconine (BHA), and benzoylmesaconine (BMA) in Fuzi should not fall below 0.010%. Known by various names including BAC, Isaconitine, and Pikraconitin, BAC appears as white crystals and exhibits solubility in methanol (8 mg/mL), ethanol, isopropanol, and trichloromethane (approximately 25 mg/mL), with slight solubility in water. Its molecular formula is C_32_H_45_NO_10_. BAC, characterized by its white crystalline form, displays notable properties. It demonstrates anti-inflammatory and analgesic effects, contributes to safeguarding cardiovascular function, and enhances glycoprotein transporter activity. Particularly in the context of cardiovascular function protection, BAC has shown significant therapeutic efficacy, as depicted in Figure 2.

## 4. Pharmacological Effects of BAC and Its Mechanisms

### 4.1. Anti-Inflammatory Effect and Mechanism

The anti-inflammatory and analgesic properties of BAC constitute its primary action, demonstrating efficacy in suppressing arthritic inflammation and various types of pain, as evidenced in ex vivo models [16]. In a study utilizing rheumatoid arthritis fibroblast-like synoviocytes (HFLS-RA) [17] (refer to Table 1), BAC inhibited the proliferation of HFLS-RA cells, showcasing in vitro antirheumatic activity. The underlying mechanism may be linked to the inhibition of inflammatory cytokine production and the downregulation of expression levels of HIF-1*α*, VEGF, and TLR4. Additionally, BAC exhibited significant anti-inflammatory effects on lipopolysaccharide (LPS)-stimulated macrophages, with the lowest effective anti-inflammatory dose compared to other MDAs [18]. *Aconiti Radix Cocta*, comprising the aforementioned MDAs, alleviated foot-plantar swelling, mitigated joint tissue inflammation and bone destruction, reduced serum levels of IL-1*β* and IL-17A, and downregulated the expression of COX-1 and COX-2 in synovial tissue in adjuvant-induced arthritis (AIA) rats [19]. Further molecular mechanism involves the regulation of arachidonic acid metabolism pathways. In another study, Gai et al. [20] encapsulated BAC into highly biocompatible copolymers to form NP/BAC for rheumatoid arthritis treatment. The NP/BAC group exhibited a 70% reduction in TNF-*α* and a 66% reduction in IL-1*β* compared to activated macrophages. This effect was attributed to the diminished overexpression of nuclear factor-*κ*B (NF-*κ*B) p65 and the inhibition of the NF-*κ*B signaling pathway. It is widely acknowledged that the content of toxic alkaloids (AC, hypaconitine (HA), and mesaconitine (MA)) decreases, while the content of nontoxic alkaloids (BAC, BHA, and BMA) increases with the prolonged decoction time of the compound formula [21]. Zhang et al. [22] demonstrated that BAC is among the constituents in the compound dHsp (derived Hei-shun-pian) that play a therapeutic role in osteoarthritis (OA).

In brief, the anti-inflammatory mechanism of BAC primarily operates by regulating the activity of inflammatory signaling pathways and diminishing the production and release of inflammatory factors such as TNF-*α* and IL-1*β*. The specific pathways involved are mostly associated with NF-*κ*B.

### 4.2. Analgesic Effect and Mechanism

In the realm of analgesia, Li, Gong, and Wang [23] observed that intrathecal administration of BAC not only effectively attenuated mechanical and thermal pain in rats afflicted with neuropathic pain but also significantly upregulated the gene expression of dynorphin in primary cultured microglia. This implies that BAC may induce analgesic effects by triggering the expression of dynorphin in spinal microglia. Despite the commendable analgesic and anti-inflammatory properties exhibited by BAC [17], its brief half-life and rapid metabolism prompted Liu et al. [24] to devise patches employing BAC as the primary ingredient. These patches, by regulating plasma drug concentrations, demonstrated efficacy in sustaining stable anti-inflammatory and analgesic effects. However, the stratum corneum's barrier function poses constraints on the transdermal penetration of BAC, ensuring the safety of drug delivery while positioning BAC as a promising candidate for inflammatory pain management [25, 26].

The analgesic mechanism of action of this substance may be linked to the modulation of central opioid receptors. Experiments have indicated that its analgesic effect can be diminished by knocking out the opioid receptor gene and administering naloxone. Furthermore, research has illustrated that the activity of the C-5's position on the aromatic ring of Fuzi alkaloids significantly impacts its analgesic efficacy [27].

### 4.3. Cardiomyocyte Protective Effect

Fuzi, with its rich history in Chinese herbal medicine and widespread usage, has garnered attention in modern pharmacological studies. Research has unveiled that numerous components within Fuzi possess the ability to enhance sinus node autoregulation, improve atrioventricular conduction, bolster cardiac pumping function, and offer protection and repair for cardiomyocytes [28, 29]. Among these components, BAC stands out as a major active ingredient in Fuzi, attracting significant attention in basic research concerning cardiovascular diseases [30, 31].

Wang et al. [32] used bioinformatics screening to explore the key molecular mechanisms and potential therapeutic agents in dilated cardiomyopathy combined with heart failure and found that BAC is a potential candidate that may exert its therapeutic effects by targeting the regulation of myocardial energy metabolism through NRK (nicotinamide riboside kinase) and NT5. Deng et al. [33] demonstrated, both ex vivo and in vivo, that BAC significantly boosts mitochondrial mass, ATP production, and the expression of proteins linked with oxidative phosphorylation (OXPHOS) complexes in HepG2 cells in a dose-dependent manner (25, 50, 75 *μ*M). Mechanistically, this enhancement is associated with the upregulation of proteins in the AMPK signaling cascade, leading to AMPK signaling activation in the heart, liver, and muscle, consequently improving cardiac function. Furthermore, Chen, Yan, and Zhang [34] (Figure 2) corroborated the modulation of the AMPK signaling pathway by BAC in their study, which simulated H9C2 cell injury in heart failure through oxygen-glucose deprivation and reperfusion (OGD/R). BAC intervention activated the AMPK/PGC-1 axis, thereby regulating mitochondrial function improvement in OGD/R-treated H9C2 cells and suppressing oxidative stress [35]. Moreover, BAC has shown promise as a potent inhibitor of erastin-induced iron death in cardiomyocytes [36]. Other studies [37] have indicated that diterpenoid alkaloids like BAC may manifest varying degrees of inhibitory effects on distinct classes of voltage-dependent potassium channel currents. These compounds have been observed to modulate the time course of cardiomyocyte action potentials, thereby potentially exerting antiarrhythmic effects.

In summary, BAC is characterized by multiple pathways and targets and is a potential protective agent for the cardiovascular system. However, most of the existing studies were conducted only at the cellular level to simulate the relevant diseases and lack of studies on primary cardiomyocytes and advanced animal models. The protective effect of BAC on cardiomyocytes may involve multiple disease intervention mechanisms at the same time, and the intrinsic connection of the pathogenesis of these several diseases may broaden the ideas for further research. On the other hand, data mining and molecular docking can be used to screen the pharmacologically active targets of BAC in the treatment of cardiovascular diseases and synergize with related pharmacodynamic studies to elucidate its pharmacological mechanism of action.

### 4.4. Alleviates the Effects of Hypertension

In another study [38], it was demonstrated that BAC exhibits the most potent blood pressure-lowering effect among several monoester alkaloids. Its administration in spontaneously hypertensive rats revealed its capability to bind to ACE/ACE2 receptors and other targets, thereby augmenting endothelium-dependent vasodilation while inhibiting ACE activity and related protein expression (Figure 2). Furthermore, BAC was found to suppress COX-2 expression and IKB-*α* phosphorylation, thereby mitigating vascular inflammation and alleviating hypertension. In summary, BAC exerts multifaceted regulatory effects on cardiovascular diseases, serving as a potential modulator of the renin–angiotensin system and a promising therapeutic agent for hypertension [39].

### 4.5. Skin Protective Effect

**Figure 3:**
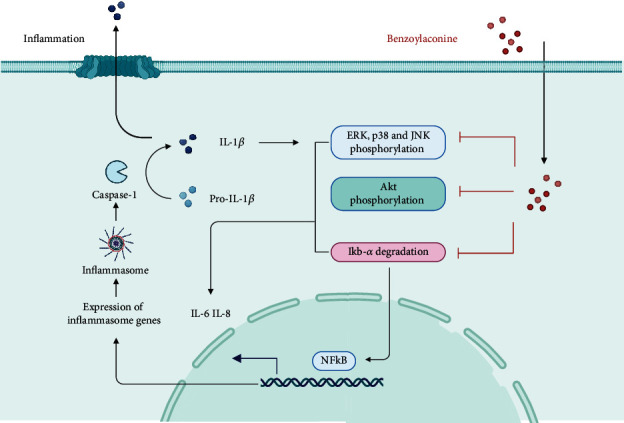
Main anti-inflammatory mechanisms of action of BAC.

Psoriasis, a chronic and multifactorial skin disease, is distinguished by inflammatory infiltration, proliferation of keratinized cells, and accumulation of immune cells [40]. In a study by Li et al., BAC was utilized to intervene in TNF-*α*/LPS-induced HaCat keratinized cells. BAC demonstrated significant efficacy in inhibiting cell proliferation and the release of inflammatory factors associated with psoriasis, without any discernible adverse effects on cell viability and safety as evidenced by ex vivo experiments. The specific mechanism of action was attributed to the suppression of the STAT3 pathway [41]. Moreover, BAC not only curtails the release of inflammatory factors such as IL-8 and IL-6 [42] but also diminishes the activation of mitogen-activated protein kinase (MAPK) and the phosphorylation of Akt. BAC predominantly exerts its protective effects on the skin by stifling the release of inflammatory factors. These actions manifest anti-inflammatory effects in IL-1*β*-stimulated human synoviocytes, and their efficacy hinges upon the MAPK, Akt, and NF-*κ*B pathways, as delineated in Figure 3.

### 4.6. Other Effects

Moreover, alkaloids like BAC notably stimulate the expression of P-glycoprotein (P-gp) and enhance exocytosis activity [43, 44]. This attribute may be attributed to its relatively mild toxicity profile. Furthermore, BAC demonstrates antidiarrheal properties by impeding spontaneous intestinal activity in mice, primarily by affecting isolated small intestinal smooth muscle [45].

## 5. Progress in Toxicological Studies

Fuzi contains a substantial amount of DDAs, with the sodium channel 2 site being the cardiotoxic target of these compounds [46]. The mechanism underlying their cardiotoxicity involves a significant influx of Na+ ions, leading to the development of persistent malignant arrhythmias. DDAs are predominantly cardiotoxic and neurotoxic, and their mechanisms of toxicity are related to their actions on voltage-dependent Na+ channels, modulation of neurotransmitter release and related receptors, promotion of lipid peroxidation, and induction of apoptosis in the heart, liver, or other organs [47, 48]. Through appropriate processing, these DDAs undergo hydrolysis at the C-8 position, resulting in their conversion to less toxic MDAs, namely, BAC, BMA, and BHA [35, 49]. When both the C-8 and C-14 groups are hydrolyzed, their derivatives become virtually devoid of toxicity, yielding nontoxic nonester alkaloids (NDAs) [50–52], as illustrated in Figure 1.

The toxicity of MDAs is significantly lower than that of DDAs, with a human lethal dose ranging from 1 to 4 mg. In mice, the toxicity of BAC is notably lower than that of DDAs, with an LD50 of 1500 mg/kg [53, 54], approximately 1/700 to 1/100 of that of DDAs. Consequently, the hydrolysis of DDAs in Fuzi produces active compounds with reduced toxicity. This process suggests the potential to discover less toxic drugs from the metabolites of DDAs, offering alternatives to the more toxic alkaloids.

In addition to this, compatibility has often been utilized to mitigate toxicity and enhance efficacy, as outlined in Table 2. When compounding Fuzi, two herbs are typically combined to achieve the dual purpose of reducing toxicity and augmenting efficacy [55]. These combinations serve as the fundamental units commonly employed in Chinese herbal formulations, offering a simpler approach compared to more intricate mixtures while preserving the basic therapeutic characteristics of Fuzi. Different combinations yield varying effects on the dissolution of Fuzi's active ingredients and metabolic enzymes within the body. Fuzi is frequently coadministered with liquorice, Ganjiang, ginseng, or paeoniae [56] to expedite the metabolism of its toxic constituents and unleash its therapeutic effects. This concurrent administration not only diminishes toxicity but also amplifies efficacy in clinical applications. The rationale behind these combinations can be elucidated by considering the drug-metabolizing enzymes of Fuzi's active ingredients and the mechanism of toxicity reduction in the combined therapy. Many of the active components in the herbs paired with Fuzi can modulate the activities of various CYP450 isoforms, countering any abnormalities in drug metabolism induced by Fuzi. For instance, Li et al. [57] observed that Fuzi exerted an inhibitory effect on hepatic microsomal CYP450 enzyme activity through hepatic microsomal CYP450 enzyme systematic assay. However, when combined with Fangfeng, a significant increase in CYP450 expression was noted. It can be inferred that the combination of Fuzi and Fangfeng induces and enhances the activity of the CYP3A4 enzyme, consequently accelerating the metabolism of DDAs, the toxic components of Fuzi.

Peng et al. conducted a study where rats were administered Fuzi and Fuzi-Ganjiang aqueous extracts, and the concentrations of MDAs and DDAs in rat plasma were determined at specified time points postadministration [58]. Compared to the Fuzi-alone group, the Fuzi-Ganjiang group exhibited reduced T1/2 and AUC_0-t_ of DDAs, alongside increased T1/2, AUC_0-t_, and Cmax of MDAs. These findings suggest that Ganjiang facilitates the clearance of DDAs while enhancing the uptake of MDAs, such as BAC. This supports the theory that the combination of Fuzi-Ganjiang reduces toxicity and enhances efficacy, providing a rationale for the Fuzi-Ganjiang combination.

In a nutshell, BAC serves as a less toxic active compound resulting from the hydrolysis of DDAs, significantly mitigating its original toxicity while maintaining its primary efficacy.

## 6. Pharmacokinetic Study of BAC

BAC is moderately soluble in water, approximately 1 mg/mL. Special solvents have also been used to aid solubilization followed by intraperitoneal administration [31, 53]. Consequently, the choice of solvents by different researchers can influence the absorption process to a certain extent. Additionally, variations in the compound's extraction processes, the percentage of Fuzi [58, 59], and the content of BAC in the final solution contribute to inconsistencies in pharmacokinetic parameters across various studies [60]. However, despite these variations, the general trends observed in the drug-time curves did not exhibit significant differences, as depicted in Table 3.

BAC demonstrates a rapid absorption rate, with blood concentration peaking within 1 h postinjection. In healthy SD rats administered 1 mg/kg of BAC via gavage, the mean maximum plasma concentration was 3.99 ± 1.20 ng/mL, and plasma concentration remained detectable within 1 h postadministration [53, 61]. BAC permeated all tissues, with concentrations peaking at 4 h. Primary accumulations were observed in the heart and kidneys, while a small fraction of BAC crossed the blood-brain barrier and was absorbed into the brain. The distribution order was as follows: heart > kidney > liver > lung > spleen > brain [53]. Similarly, Xiaojun et al. investigated the distribution of several key alkaloids in various tissues and organs of rabbits after administering a high dose of aqueous aconite extract (0.20 ± 0.05 mg/g) via gavage. Their study revealed that BAC was highly concentrated in the spleen, liver, and kidney tissues of the rabbits [62].

The cardioprotective effect of BAC is currently its most recognized efficacy. Zhou et al. [63] introduced a novel method employing ultraperformance liquid chromatography-mass spectrometry (UPLC-MS/MS) and applied it to the pharmacokinetic study of three MDAs in normal and myocardial infarcted rats after the oral administration of SND (Sini decoction). The study revealed that following the oral administration of SND, myocardial infarction rats exhibited lower blood concentrations of the MDAs compared to normal rats. Additionally, the elimination and distribution of these alkaloids were slower in myocardial infarction rats, resulting in less systemic exposure, nontoxicity, and evident cardioprotective effects. In vivo distribution studies of BAC also indicated a higher concentration of the drug in the heart, providing supportive evidence for its pharmacodynamic effects.

Plasma proteins serve a pivotal role in enhancing the pharmacokinetic properties of drugs by aiding in their transport and distribution throughout the body via the bloodstream. Thus, Zhou et al. [64] employed multispectroscopy, molecular docking, and kinetic simulation to explore the interaction mechanism between human serum albumin (HSA) and BAC. The study revealed that residues such as TRP-214, LEU-219, LEU-238, and ALA-291 played crucial roles in the binding of BAC to HSA, thereby shedding light on the distribution and metabolic pattern of BAC.

**Figure 4:**
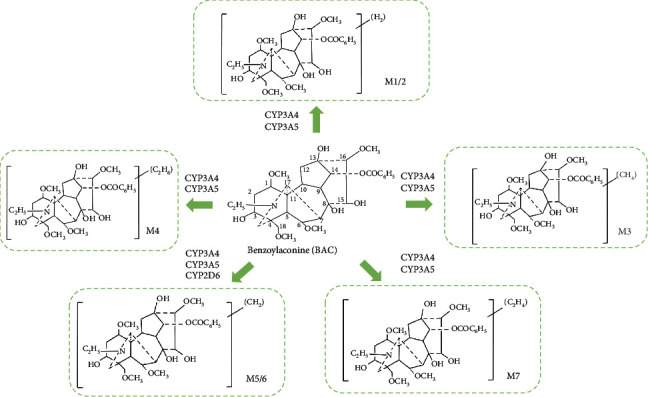
Major metabolites of benzoylaconitine.

BAC is primarily metabolized by CYP3A4 and CP3A5 [65], and the metabolic pathways include demethylation, dehydrogenation, hydroxylation, and dimethylation. Regarding the determination of metabolic composition, the unique structural features of BAC result in a greater number of metabolites but with lower concentrations, posing challenges for structural identification. Seven known metabolites include dehydrogen-BAC, dehydrogen-BAC, demethyl-dehydrogen-BAC, didemethyl-dehydrogen-BAC/deethyl-dehydrogen-BAC, Demethyl-BAC, demethyl-BAC, and deethyl-BAC/didemethyl-BAC, and the metabolic process is shown in Figure 4. It is evident that the metabolic pathway of BAC is largely mediated by CYP3A, implying that caution should be exercised in the clinical use of Fuzi in combination with drugs metabolized by CYP3A4, as there is a potential for drug-drug interactions. BAC is predominantly excreted through feces, with the fecal concentration of BAC being considerably higher than that in urine.

Noteworthy is a pharmacokinetic study conducted in humans [66]. Three dosages at low (10.0 mg/kg), medium (13.3 mg/kg), and high (16.7 mg/kg) levels of “SHEN-FU” injectable powder were applied on 18 healthy volunteers by intravenous drop infusion. Every 10 mg “SHEN-FU” injectable powder contained 1103.5 ng BAC. Six volunteers were involved in each experiment. The results indicated that the half-life of BAC at all three administered doses was approximately 1 h. They all achieved the maximum concentration at 30 min in medium dosage, however, 45 min in low and high dosages.

## 7. Discussion and Future Perspectives

This review represents the first comprehensive summary of the research progress regarding the pharmacological effects of BAC, a less studied and less toxic component of Fuzi. BAC demonstrates promising potential for development into an effective clinical formulation. Recent studies on BAC have emphasized chemical spectrum analysis, pharmacological investigations, conventional toxicological studies, and its potential therapeutic value [67, 68]. BAC exhibits diverse effects in the treatment of cardiovascular diseases, anti-inflammatory and analgesic properties, and the regulation of immune function, showcasing its involvement in disease treatment through multiple targets and pathways. In relation to cardiovascular disease, there is a burgeoning body of research indicating the significant involvement of inflammatory factors [69]. Oxidative stress and inflammation stand out as major contributors to cardiovascular risk factors such as hypertension and dyslipidemia. Therefore, mitigating systemic inflammation holds promise for decreasing the likelihood of cardiovascular events. A study demonstrated that decreased levels of IL-6 led to a 32% reduction in the risk of major adverse cardiovascular events [70]. The potent anti-inflammatory properties of BAC underscore its potential as a cardioprotective agent.

Regarding the mode of BAC administration and dosage, which may be responsible for its lower bioavailability and faster elimination, frequent intravenous injections of about 2–10 mg/kg (Table 1) were used in most of the animal experiments, and there was a clear dose dependence of BAC in the safe range (LD_50_ of 1500 mg/kg) [53].

Nevertheless, there remain several aspects of BAC research that warrant further exploration. Firstly, the safety, tolerance, and efficacy of BAC are not yet fully understood, and its clinical value, particularly regarding potential toxic side effects [71], necessitates further verification based on current studies to facilitate its clinical application. Secondly, the specific mechanism of BAC's action remains elusive. Hence, it is imperative to investigate the multitarget, multipathway pharmacokinetic-pharmacodynamic interactions of BAC to uncover the intrinsic science behind its therapeutic effects. Given that BAC is rapidly absorbed in the body, understanding its metabolic processes is crucial. For example, the pharmacokinetic parameters Cmax, Tmax, and T1/2 of BAC were found to be 39.85 ± 12.02 ng/mL, 0.31 ± 0.17 h, and 9.49 ± 0.49 h, respectively. These values suggest that BAC is quickly absorbed but not easily metabolized or excreted. However, the lower Cmax indicates that the bioavailability of BAC is not high, potentially due to inhibition by some transport proteins [68]. Future research on BAC should explore its metabolites, their related ratios, and comprehensively study the pharmacological effects and toxicities of BAC to provide a reliable foundation for its clinical application.

Additionally, DDAs significantly restrict the utilization of Fuzi [72]. While BAC's pharmacological action as an active ingredient holds promise in alleviating this limitation, its extraction technology poses challenges, and the extraction cost is high [73, 74]. Addressing these issues requires further basic research and technical support.

Another aspect worth noting is the low bioavailability of BAC, which has significantly restricted the development and clinical application of BAC-related formulations. Therefore, exploring new delivery systems to enhance the bioavailability of BAC presents an intriguing research direction. Overall, research on the pharmacological mechanism of action of BAC and Fuzi is gradually deepening, particularly in the field of cardioprotection [75], which is of significant interest. However, there remains a lack of clinical trials to confirm the safety and efficacy of BAC. The complex extraction process and expensive production cost of BAC, combined with the limited number of current studies on its pharmacological effects, have posed barriers to its clinical application. Some researchers have incorporated Fuzi as the main ingredient into injections and conducted clinical studies on its efficacy [76, 77], yielding positive results. It is anticipated that the mechanism of action of BAC will become clearer with further deepening of research.

## Figures and Tables

**Table 1:** Basic information on experiments related to the pharmacological effects of BAC and its mechanisms.

**Refs**	**Research model**	**Phenotype/pathways**	**BAC dose (way)**	**Effect**
*Anti-inflammatory effect and mechanism*				
[17]	HFLS-RA cell	Inhibition of inflammatory cytokine production	1000 *μ*g/mL	TLR4, HIF-1*α*, and VEGF↓
[20]	Rheumatoid arthritis mice LPS-induced RAW264.7	Inhibition of inflammatory cytokine production	Mice 10 mg/kg, iv injection Cell: 5, 18, and 40 *μ*g/mL	NF-*κ*B p65↓ IL-1*β*, and TNF-*α*↓
[22]	Osteoarthritis rat	Inhibits chondrocyte hypertrophy	14 g/kg dHSP, orally administered for 28 days	Col2↑ Col10, Mmp2, and Sox5↓
[42]	LPS-activated RAW264.7 macrophage cell	MAPK and NF-*κ*B pathways	500 *μ*M	IL-6, TNF-*α*, IL-1*β*, ROS, NO, and PGE2↓ iNOS, COX-2↓
*Analgesic effect and mechanism*				
[23]	Spinal L5/L6 nerve-ligated neuropathic rats	Attenuated mechanical allodynia and heat hyperalgesia	2.2 *μ*g, intrathecal injection	Dynorphin A↑
[24]	Acetate pain mouse model	Decreased the writhing number significantly	350 *μ*g/cm^2^; 5.0 cm^2^	Pain inhibition ratio > 50%
*Cardiomyocyte protective effect*				
[33]	HepG2 cell Balb/c mice	AMPK signaling	25, 50, and 75 *μ*M 10 mg/kg per day, for 7 days, ip	OXPHOS, mtDNA, ATP, and mitochondrial mass↑
[34]	OGD/R-induced cardiomyocyte injury	AMPK/PGC-1 axis	25, 50, 75, 100, and 125 *μ*M	p-AMPK, PGC-1*α*↑
*Alleviates the effects of hypertension*				
[38]	Spontaneously hypertension rats	ACE/ACE2; Akt/eNOS	0.6, 2, and 6 mg/kg; iv injection	NO↑ Ang II, TNF-*α*, IL 6, COX-2, and IKB*α*↓
*Skin protective effect*				
[41]	TNF-*α*/LPS-induced HaCaT keratinocytes	STAT3 pathway	10, 20, and 40 *μ*M	TNF-*α*, IL-17, and p-STAT3↓

Abbreviations: ACE, angiotensin-converting enzyme; AMPK, adenosine 5′-monophosphate (AMP)-activated protein kinase; COX-2, cyclooxygenase 2; HFLS-RA, rheumatoid arthritis; LPS, lipopolysaccharide; OGD/R, oxygen-glucose deprivation and reperfusion; PGE2, prostaglandin E2; ROS, reactive oxygen species; STAT3, signal transducer and activator of transcription 3; TLR4, toll-like receptor 4; TNF-*α*, tumor necrosis factor *α*; VEGF, vascular endothelial growth factor.

**Table 2:** Classical Chinese herbal medicine compound of Fuzi.

**Year Refs**	**Herbal combination**	**Ratio of compounds**	**Coadministration effect**
2013 [58]	Fuzi-Ganjiang	1 : 1	Ganjiang could promote the elimination of AC and HA and enhance the absorption of BAC.
2017 [61]	Fuzi and Beimu	1 : 1	Fuzi in combination with Beimu was favorable for absorption of BMA and BHA.
2017 [60]	Fuzi-Mahuang	11 : 6	Decreased cumulative excretion of monoester alkaloids (BAC and BHA)
2022 [59]	Fuzi and ginseng	1 : 1	Could significantly increase the bioavailability and efficiency of active components in vivo
2015 [28]	Shenfu decoction	1 : 1	Shenfu decoction could significantly improve hepatic injury in CHF patients.
2018 [29]	Shenfu formula	1 : 1	Shenfu granule can effectively improve cardiac function in heart failure rats.

**Table 3:** BAC main pharmacokinetic parameters.

**Year Refs**	**Subjects**	**Dose**	**BAC parameters**			
			**T1/2 (min)**	**Tmax (min)**	**Cmax (ng/mL)**	**AUC**_**0−t**_ **(ng/min/mL)**
2013 [58]	Rats (*n* = 6)	5.4 g crude drug of Fuzi/kg	186.62 ± 14.12	35.18 ± 5.88	1.16 ± 0.05	355.67 ± 19.43
2016 [53]	Rats (*n* = 6)	1 mg/kg BAC	9.49 ± 0.49 h	0.31 ± 0.17 h	3.99 ± 1.20	13.54 ± 2.29 (ng/h/mL)
2017 [61]	Rats (*n* = 6)	Fuzi aqueous, 20 g/kg	5.67 ± 1.49 h	1.93 ± 1.26 h	2.83 ± 0.73	18.96 ± 5.82 (*μ*g·h/L)
2019 [63]	Rats (*n* = 6)	18.95 mg/kg BAC	12.38 ± 4.02 h	0.71 ± 0.13 h	12.82 ± 5.80	40.44 ± 13.61 (ng/h/mL)
2022 [59]	Rats (*n* = 6)	2 g/kg of Fuzi	9.28 ± 0.18 h	1.00 ± 0.07 h	0.39 ± 0.22	4.47 ± 0.54
2014 [78]	Rats (*n* = 6)	18.75 *μ*g/mL–1.5 mL/100 g	9.40 ± 2.30 h	0.60 ± 0.30 h	151.60 ± 129.30	323.80 ± 190.80 (ng/h/mL)

## Data Availability

The data supporting this review are from previously reported studies and datasets, which have been cited. The processed data are available from the corresponding author upon request.
